# LiFePO_4_/C twin microspheres as cathode materials with enhanced electrochemical performance†

**DOI:** 10.1039/d3ra00183k

**Published:** 2023-03-01

**Authors:** Yiqiong Peng, Lingzhi Zeng, Shuai Dai, Feng Liu, Xi Rao, Yongping Zhang

**Affiliations:** a School of Materials and Energy, Southwest University Chongqing 400715 China zhangyyping6@swu.edu.cn raoxiemail@swu.edu.cn

## Abstract

Self-assembled lithium iron phosphate (LiFePO_4_) with tunable microstructure is an effective way to improve the electrochemical performance of cathode materials for lithium ion batteries. Herein, self-assembled LiFePO_4_/C twin microspheres are synthesized by a hydrothermal method using a mixed solution of phosphoric acid and phytic acid as the phosphorus source. The twin microspheres are hierarchical structures composed of primary nano-sized capsule-like particles (about 100 nm in diameter and 200 nm in length). The uniform thin carbon layer on the surface of the particles improves the charge transport capacity. The channel between the particles facilitates the electrolyte infiltration, and the high electrolyte accessibility enables the electrode material to obtain excellent ion transport. The optimal LiFePO_4_/C-60 exhibits excellent rate performance with discharge capacity of 156.3 mA h g^−1^ and 118.5 mA h g^−1^ respectively at 0.2C and 10C, and low temperature performances with discharge capacity of 90.67 mA h g^−1^ and 66.7 mA h g^−1^ at −15 °C and −25 °C, respectively. This research may provide a new pathway to improve the performance of LiFePO_4_ by tuning the micro-structures by adjusting the relative content of phosphoric acid and phytic acid.

## Abstract 2

Schematic illustration of self-assembled twin microspheres of LiFePO4/C: connection mode and structure.

## Graphical abstract

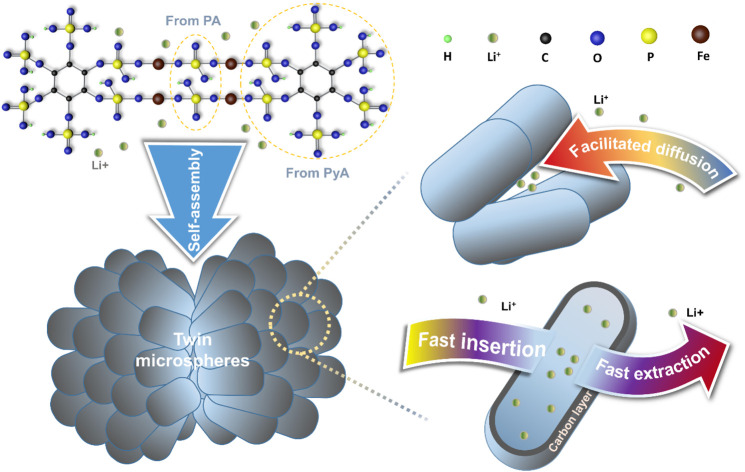

## 1. Introduction

With the rapid development of the electric vehicle industry, the demand for lithium ion batteries with high-capacity, high-speed performance and higher safety has been increasing. Lithium iron phosphate (LFP) has become one of the most flourishing commercial cathode materials for lithium ion batteries due to its high theoretical specific capacity (170 mA h g^−1^), low cost, high stability and environmental friendliness.^1^ However, the electrochemical performance of LiFePO_4_, especially the rate performance and low temperature performance, has been limited by some shortcomings, including: (1) the large LiFePO_4_ particles synthesized by the conventional method resulted in a longer lithium ion transport path and lower effective contact area between the active material and electrolyte.^2^ (2) Low electronic conductivity limited high-rate capability of lithium iron phosphate.^3^ (3) Weak Li^+^ diffusivity deteriorated the electrochemical performance.^4–6^ Certain strategies were proposed to tackle these problems: firstly, shortening the lithium ion migration path and increasing the contact area between the active material and the electrolyte by particle nanocrystallization.^7–9^ Secondly, coating conductive material to enhance the conductivity of the electrode material. The most commonly applied method was to coat carbon layers to cover the electrode material and build a three-dimensional conductive network.^10–15^*In situ* carbon coating reduces the particle size of LiFePO_4_ by inhibiting the growth of particles during sintering. Carbon in high temperature environment was used as a reducing agent to inhibit the oxidation of divalent iron during sintering. Uniform carbon coating also protected active material avoiding HF corrosion and surface degradation.^16^ Thirdly, cation doping can improve the intrinsic conductivity of electrode materials and broaden Li^+^ diffusion channel by changing the lattice structure of LiFePO_4_.^17–22^ In practical applications, nanostructure formation and carbon coating were combined to improve both electronic conductivity and ion transport. However, the thermodynamic instability and the probability of side reactions were greatly increased when the particle of LiFePO_4_ reduced to nanoscale size, and the machinability and tap density are reduced.^23^ Adding too much carbon material will also reduce the tap density of LiFePO_4_/C composites. Therefore, LiFePO_4_/C particles with low carbon content and assembled into a hierarchical structure thereby provided higher tap density and volumetric energy density. Fabrication of self-assembled hierarchical electrode materials by adjusting microstructure has proven to be an effective method for improving stability and kinetic activity.^23–28^

Hydrothermal and solvothermal synthesis techniques were commonly used to fabricate LiFePO_4_/C composites with hierarchical structure. While the solvothermal method was unsuitable for large-scale production, because it requires relatively expensive organic solvents.^29–31^ Hydrothermal synthesis was often combined with surfactants to construct special morphological structures. These surfactants are usually lost during washing step or separated as impurities.^32,33^ Phytic acid was widely used as structure-directing agent and a reaction raw material in recent years. Phytic acid is a natural organic compound containing a six-membered carbon ring and six phosphate groups. Due to the negative charge on its surface, it can chelate a variety of metal cations. Phytic acid was utilized for the structural orientation of LiFePO_4_ self-assembly synthesis.^34^ At the same time, it is retained in LiFePO_4_ products as a phosphorus source for the synthesis reaction. A lot of researches were carried out to improve the charge–discharge performance of LiFePO_4_ by using phytic acid as a phosphorus source. Cao *et al.* prepared glucose-derived carbon and graphene co-modified LiFePO_4_ composite microspheres with phytic acid as a phosphorus source, and proved the important role of phytic acid in the construction of self-assembled structure.^28^ Zhao *et al.* introduced phytic acid-derived internal carbon sheets using phytic acid as a phosphorus source, resulting in an ultra-high capacity of 192 mA h g^−1^ and excellent rate performance.^26^ Li and his colleagues connected Fe^2+^ and carbon nanotubes through the special functional groups of phytic acid to form a three-dimensional network around LiFePO_4_ particles, which improved the conductivity and ion transport capacity of LiFePO_4_/C composites.^35^ But these works often used a large amount of inorganic carbon materials such as graphene and carbon nanotubes, while too much carbon content reduced the tap density of materials. Therefore, we preferred to use a simple hydrothermal method to prepare LiFePO_4_/C composites with low carbon content and high tap density, to achieve excellent electrochemical performance improvement through microstructure adjustment.

Herein, twin microspheres LiFePO_4_/C electrode materials were prepared by hydrothermal method with phosphoric acid and phytic acid as phosphorus sources. LiFePO_4_/C twin microspheres were composed of capsule-like LiFePO_4_ nanoparticles with uniform size. The thin carbon layer was uniformly covered on the surface of the nanoparticles, which effectively improves the electronic conductivity and lithium ion conductivity of LiFePO_4_.

## 2. Experimental section

### 2.1 Chemicals

Ferrous sulfate heptahydrate (FeSO_4_·7H_2_O, 99%), phytic acid (PyA, 70%), phosphoric acid (PA, 85%), lithium hydroxide monohydrate (LiOH·H_2_O, 99%), glucose (C_6_H_12_O_6_, 99%) were purchased from Aladdin Biochemical Technology Co., Ltd (Shanghai, China).

### 2.2 Synthesis of LiFePO_4_/C

**Fig. 1:**
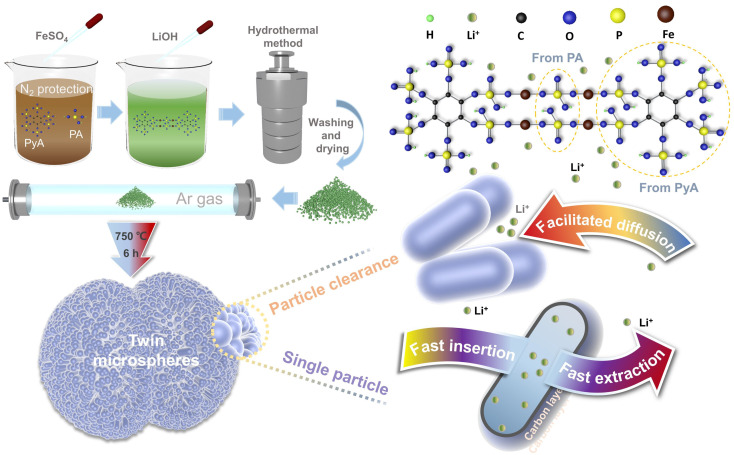
Schematic illustration of self-assembled twin microspheres LiFePO_4_/C cathode materials.

LiOH·H_2_O, FeSO_4_·7H_2_O and phosphorus source (PA, PyA) were weighed according to the molar ratio of Li : Fe : P = 3 : 1 : 1.1. Phytic acid and phosphoric acid were dissolved in deionized water with the proportion of phosphoric acid to phytic acid of 0%, 40%, 60%, 80% and 100%, respectively. The obtained brown mixed solution was bubbling with high-purity nitrogen for 30 minutes. Ferrous sulfate was dissolved in deionized water and then quickly added to the above mixed solution under stirring. Then, LiOH solution was slowly added to obtain a dark green suspension. The preparation process and connection mode of twin microspheres LiFePO_4_/C composites were schematically illustrated in Fig. 1. Phytic acid molecule chelated the iron atom, and the phosphoric acid acted as a bridge to coordinate with the phytic acid to form a core of LiFePO_4_. LiOH solution was used as precipitant to obtain suspension. The suspension was stirred at a constant speed for 30 minutes and then transferred to a stainless steel autoclave lined with polytetrafluoroethylene. The reaction was carried out at 210 °C for 12 h and naturally cooled to room temperature. The precipitate was repeatedly washed with deionized water and dried at 60 °C for 8 h to obtain a gray-green precursor. The gray-black composite was obtained by adding 5 wt% glucose and fully grinding, annealing at 750 °C for 6 h in a tube furnace under Ar environment. The products were marked as LFP/C-0, LFP/C-40, LFP/C-60, LFP/C-80 and LFP/C-100 accordingly.

### 2.3 Characterization

Morphological and structural investigations were carried out with X-ray diffraction (XRD, SHIMADZU XRD-7000, Cu Kα radiation *λ* = 1.54178 Å), field-emission scanning electron microscopy (JEOL, JSM-7800F) coupled with an energy dispersive X-ray spectrometer (EDS) and transmission electron microscope (JEOL, JEM-2100F). X-ray photoelectron spectroscopy (XPS, ULVCA-PHI, PHI5000) was used to characterize the chemical states. Raman spectra were performed on the HORIBA LabRAM HR Evolution instrument with an excitation laser wavelength of 532 nm. The DSC-TGA analysis was performed on a TGA/DSC1/1600LF device, heated in a temperature range of 25–700 °C with a heating rate of 10 °C min^−1^ in the flowing air environment. BET specific surface areas, and the pore size distributions were determined by a JW-BK300 analyzer.

### 2.4 Electrochemical measurement

Active materials, acetylene black (ACET) and polyvinylidene fluoride (PVDF) binder were dispersed in *N*-methylpyrrolidone (NMP) at a weight ratio of 8 : 1 : 1. The viscous slurry coated on aluminum foil and dried in a vacuum oven at 80 °C overnight to obtain working electrode. The active substance loading was about 2.0 mg cm^−2^. The CR2032 coin-type half-battery was assembled in a glove box filled with argon. The lithium foil and celgard 2400 polypropylene microporous film were used as the anode electrode and separator respectively. The electrolyte was prepared by dissolving 1 M LiPF_6_ into a mixture of ethylene carbonate, dimethyl carbonate and methyl ethyl carbonate with a volume ratio of 1 : 1 : 1 (EC : DMC : MC = 1 : 1 : 1). The battery test system (Wuhan LAND, CT2001A) is used to fulfil Galvanostatic charge/discharge tests of the assembled battery. Electrochemical impedance spectroscopy (EIS) and cyclic voltammetry (CV) were conducted on an electrochemical workstation (Chenhua, CHI750E). All electrochemical measurements were performed in a fixed voltage window between 2.4–4.2 V (*vs.* Li/Li^+^) at room temperature.

## 3. Results and discussion

### 3.1 Morphology and structural characterization

**Fig. 2:**
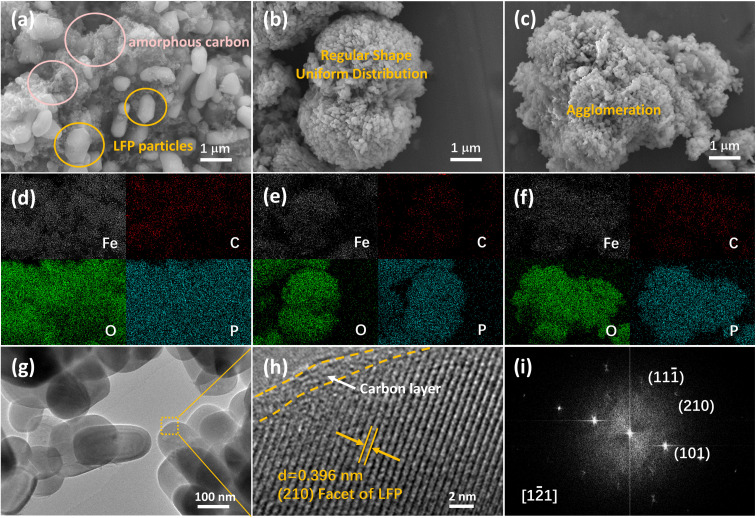
SEM images (a, b and c) and corresponding EDS mapping (d, e and f) of LFP/C-0, LFP/C-60, LFP/C-100; TEM (g), HRTEM (h) and FFT image of LFP/C-60.

Morphology of LiFePO_4_/C composites was observed by field emission scanning electron microscopy (FESEM) and transmission electron microscopy (TEM). FESEM images and corresponding EDS mapping of LFP/C-0, LFP/C-60 and LFP/C-100 samples are presented in Fig. 2(a–f). FESEM images of LFP/C-40 and LFP/C-80 and the corresponding EDS mapping are shown in Fig. S1.† LFP/C-0 sample in Fig. 2(a) appears as a large particle size (0.8–1.2 μm), polyhedral particle edges are clearly distinguishable, and the carbon coating is uneven. SEM image in Fig. S1(a)† shows that LFP/C-40 exhibits the polyhedron structure with reduced size, the edges and corners are smoothed, and a small number of twin microspheres structures are observed. In Fig. 2(b), the twin microspheres LFP/C-60 were formed by the aggregation of capsule-like particles with a diameter of 100 nm. SEM images in Fig. S2† showed that most of the LiFePO_4_/C-60 samples appeared as twin microsphere structures with slightly different size. Twin microsphere structures were formed at suitable ratio of phytic acid and phosphoric acid. This arrangement made it possible for LiFePO_4_/C composites to obtain ideal bulk density while refining grains. Uniform carbon coating forms a conductive network in the twin microspheres structure, which increase the electronic conductivity. The channels between the particles and the large specific surface area allow the electrolyte to be fully wetted, facilitating the transport of ions through the electrode, which improved the electrochemical performance of the electrolytic material. The twin microspheres structure was also observed in LFP/C-80. Compared with LFP/C-60, the bonding between primary particles tensed and the gap between particles was occupied. FESEM image in Fig. 2(c) depicted LFP/C-100 appears as large aggregated clusters. With the increase of phytic acid content, the primary particle size became smaller, but excessive introduction of phytic acid would lead to serious aggregation between particles. When the bonding degree increased, the gap between the primary particles was blocked, forming a larger particle size block, which inhabited electrolyte infiltration and ion transmission. This indicates that phytic acid is pivotal to self-assembly and fusion between particles, while phosphoric acid is related to the core connection of two hemispheres. The morphological characteristics can be regulated by the ratio of phytic acid to phosphoric acid. In addition, the EDS mappings of LFP/C-40 and LFP/C-80 show that the carbon coating is uniform. Compared with LFP/C-0, the self-assembled structure is not difficult to achieve uniform carbon coating. Fig. 2(g) shows the TEM image of LFP/C-60, which shows short rod-shaped primary particles with reliable connection between particles. High-resolution transmission electron microscopy (HRTEM) and fast Fourier transform (FFT) images in Fig. 2(h and i) shows clear and distinguishable lattice fringes for LFP/C-60. LFP/C-60, reflecting the properties of its single crystal. The interplanar spacing is 0.395 nm, corresponding to the (210) crystal plane of the orthogonal phase LiFePO_4_. A thin amorphous carbon layer of about 2 nm forms a conductive network on the surface of LiFePO_4_, which provides a channel for electron transport, significantly improves the electron transport capacity of the particles and inhibits the further growth of the particles. The lattice spacing and the corresponding FFT images verified the main exposed crystal plane of LFP/C-60 is (210).

**Fig. 3:**
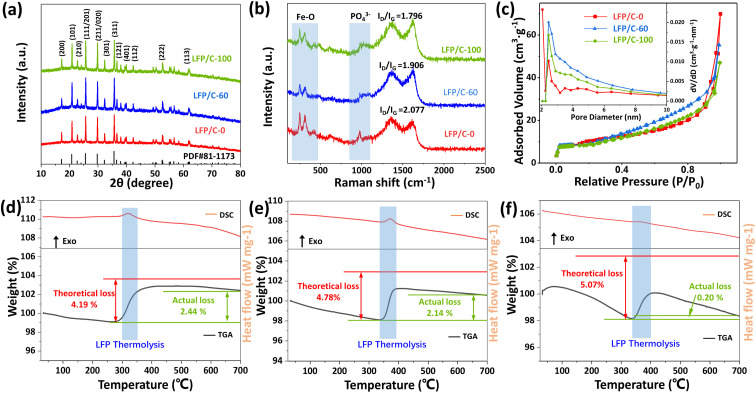
XRD patterns (a), Raman spectra (b), and N_2_ adsorption–desorption isotherms and BJH pore size distribution plots (c) of LFP/C-0, LFP/C-60, LFP/C-100. TG-DSC curves (d, e and f) of LFP/C-0, LFP/C-60, LFP/C-100 recorded from room temperature to 700 °C at a heating rate of 10 °C min^−1^ in air.

Fig. 3(a) shows the XRD patterns of LFP/C-0, LFP/C-60 and LFP/C-100. The main diffraction peaks of the three samples are consistent with JCPDS-081-1173, belonging to the orthorhombic system. The diffraction peaks of the samples are sharp, indicating that the LiFePO_4_/C composites have high crystallinity. An inconspicuous peak assigned to the carbon is observed in the range of 15°–35° due to its low coated carbon content or amorphous state, and the peak is obscured by the strong peak of lithium iron phosphate.^36,37^ In order to determine the microcrystalline orientation of LiFePO_4_, the peak intensity ratio of *I*_(200)_/*I*_(020)_ was calculated according to the XRD pattern. The LFP/C-0, LFP/C-60, and LFP/C-100 ratios were 0.309, 0.325, and 0.368, respectively. This indicates that phytic acid is beneficial to the preferential growth of LiFePO_4_ along the (100) crystal plane. The lattice parameters of LFP/C-60 are determined to be *a* = 6.008 Å, *b* = 10.334 Å and *c* = 4.693 Å, respectively, which are in good agreement with the theoretical value (*a* = 6.01 Å, *b* = 10.332 Å and *c* = 4.692 Å). The grain size of LFP/C-60 calculated by Scherrer equation is about 90.4 nm, which is consistent with the FESEM observation results.

Fig. 3(b) depicts the Raman spectra of LFP/C-0, LFP/C-60 and LFP/C-100 samples. The bands at 100–500 cm^−1^ and 520–1120 cm^−1^ correspond to the characteristic peaks of Fe–O and PO_4_^3−^ in LiFePO_4_, respectively.^38^ The two clear characteristic peaks indicates that the carbon coating is so thin that the probe laser beam can pass through, which is consistent with the observation results of transmission electron microscopy. The peak intensity at 980 cm^−1^ of LFP/C-60 and LFP/C-100 is weaker than that of LFP/C-0, which indirectly indicates that uniform carbon coating is achieved and the signal of PO_4_^3−^ is partially shielded. The bands at 1350 cm^−1^ and 1625 cm^−1^ correspond to the D (disordered carbon, sp^3^) and G (graphite, sp^2^) peaks of amorphous carbon.^39,40^ The peak area ratio of D and G (*I*_D_/*I*_G_) usually provides a useful index for comparing the crystallinity of various carbon materials. The smaller the *I*_D_/*I*_G_, the higher the order degree of carbon materials. *I*_D_/*I*_G_ ratio of LFP/C-0, LFP/C-60 and LFP/C-100 is 2.08, 1.91 and 1.80, respectively. With the increase of the proportion of phytic acid, the graphitization extent of carbon in the material is also increased, which indicates that the derived carbon of phytic acid is more easily graphitized during annealing. To verify above conclusion, the precursor of pure phytic acid group was annealed and carbonized directly in argon at the same temperature to obtain LFP-100, in which the carbon was phytic acid derived carbon. The Raman spectrum of LFP-100 (Fig. S3†) shows that the sample has *I*_D_/*I*_G_ = 1.263 and the highest degree of graphitization. Owing to the absence of amorphous carbon layer, the Raman signal near 980 cm^−1^ shows a strong and sharp peak.

The pore size distribution and nitrogen adsorption–desorption curves of LFP/C-0, LFP/C-60 and LFP/C-100 were illustrated in Fig. 3(c). BET specific surface area is 34.18 m^2^ g^−1^, 34.24 m^2^ g^−1^, and 32.57 m^2^ g^−1^, for LFP/C-0, LFP/C-60 and LFP/C-100, respectively. The pore volume is about 0.090 cm^3^ g^−1^, 0.085 cm^3^ g^−1^ and 0.069 cm^3^ g^−1^, for LFP/C-0, LFP/C-60 and LFP/C-100, respectively. The adsorption–desorption isotherms show that LFP/C-60 exhibits a typical H4 type hysteresis loop in the relative pressure range of 0.8–1, suggesting the presence of massive mesopores resulting from the stacking of nanoparticles.^41^ The mesopores makes LFP/C-60 electrode material to better adsorb electrolyte during charge and discharge, thereby enhancing ion conductivity. The pore size distribution of the three samples is concentrated in the range of 2–10 nm, with a peak at near 2.5 nm.

The carbon content of LFP/C-0, LFP/C-60 and LFP/C-100 was measured by thermogravimetric analysis, and the DSC-TGA curves are shown in Fig. 3(d–f). LiFePO_4_ began to be oxidized to Li_3_Fe_2_ (PO_4_)_3_ and Fe_2_O_3_ at about 350 °C, resulting in a mass increase. The reaction formula of pure LiFePO_4_ in air is shown in Formula (1).^42^ Carbon burns at about 430 °C, corresponding to a mass loss. The carbon content in LFP/C-0, LFP/C-60, LFP/C-100 was calculated to be 1.75 wt%, 2.64 wt% and 4.87 wt%, respectively, according to Formula (2).^35^

1 LiFePO_4_ + 1/4 O_2_ = 1/3 Li_3_Fe_2_(PO_4_)_3_ + 1/6 Fe_2_O_3_

2 % carbon = theoretical weight gain of LiFePO_4_ − total weight gain of the composites

**Fig. 4:**
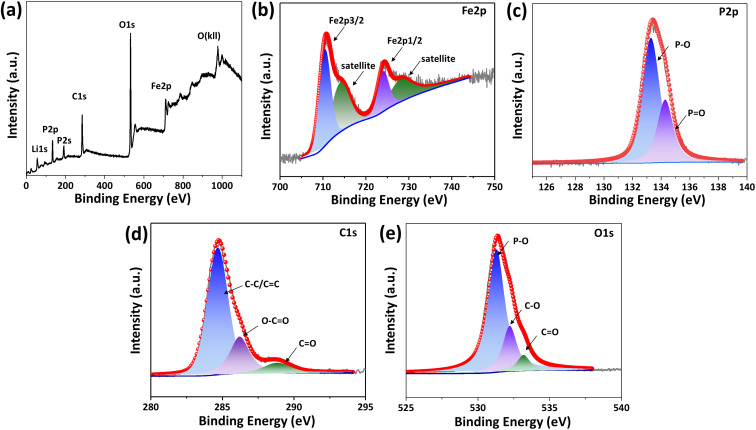
XPS survey (a), high resolution XPS spectra of Fe 2p (b), P 2p (c), C 1s (d), and O 1s (e) for LFP/C-60.

X-ray photoelectron spectroscopy (XPS) survey spectrum in Fig. 4(a) represents that peaks situated at 711.1 eV, 531.5 eV, 284.8 eV, 192.5 eV, 133.8 eV, and 55.9 eV are ascribed to Fe 2p, O 1s, C 1s, P 2s, P 2p, and Li 1s, respectively for LiFePO_4_/C composite. High-resolution XPS Fe 2p spectrum in Fig. 4(b) shows peaks at 710.6 eV and 724.1 eV are attributed to Fe 2p_3/2_ and Fe 2p_1/2_, respectively, with spin–orbit splitting of 13.5 eV, which is consistent with the previously reported spectra of Fe^2+^ in LiFePO_4_.^37,43^ Peaks at 713.9 eV and 727.4 eV are identified as satellite peaks. The characteristic peaks of P–O and P=O appear at 133.3 eV and 134.3 eV in the P 2p spectrum of Fig. 4(c), corresponding to the presence of phosphate. The C 1s peak at 284.6 eV corresponds to C–C, and the peaks at 286.0 and 288.9 eV correspond to C=O and O–C=O, respectively, as shown in Fig. 4(d). The O 1s spectrum shown in Fig. 4(e) shows a peak at 531.3 eV, which confirms that the divalent state of O exists in LiFePO_4_/C-60. The peaks at 532.3 eV and 533.2 eV correspond to the C–O and C=O functional groups absorbed on the surface of the product, which may be related to the interface bonding between the carbon layer and LiFePO_4_.

### 3.2 Electrochemical characterization

**Fig. 5:**
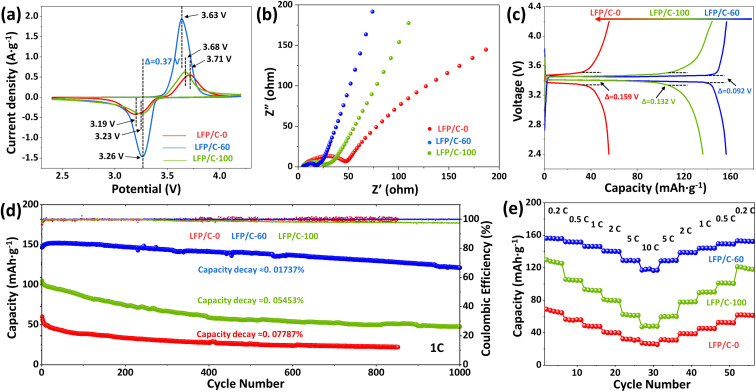
The electrochemical performance of LFP/C-0, LFP/C-60, LFP/C-100: (a) CV curves with scanning rate of 0.5 mV s^−1^; (b) electrochemical impedance spectra (EIS); (c) initial charge/discharge curves at 0.2C; (d) cycle performances at 1C; (e) rate performances of various discharge rates between 0.2C and 10C.

Cyclic voltammetry (CV) was used for testing phase transition and ionic conductivity. Fig. 5(a) shows the cyclic voltammetry curves of LFP/C-0, LFP/C-60 and LFP/C-100 as cathode active materials at 0.5 mV s^−1^ scan rate, and the voltage window is 2.4–4.2 V. All samples show an oxidation peak and a reduction peak related to the conversion of Fe^3+^ and Fe^2+^, corresponding to the insertion and extraction of Li^+^ in LiFePO_4_. LFP/C-60 exhibits the largest peak area, indicating the highest specific capacity among other samples. Judging from the shape of peak, the redox peak of LFP/C-60 is more sharp and symmetrical, indicating that LFP/C-60 has better reaction kinetics and reversibility. Potential interval of oxidation peak and reduction peak can reflect polarization characteristics of electrode materials.^44^ The redox potential interval of the three samples is 0.52 V, 0.37 V and 0.45 V, respectively. Obviously, the polarization of LFP/C-60 is the lowest. Lower electrode polarization indicates an excellent electrochemical conversion reaction between Fe^3+^ and Fe^2+^ during Li^+^ insertion/extraction, owing to the increased contact area between the electrolyte and the electrode and the convenience of the Li^+^ insertion/extraction process.

Electrochemical impedance spectroscopy (EIS) in Fig. 5(b) represents the Nyquist plots of three LiFePO_4_/C composites, which are composed of semicircles in the intermediate frequency region and straight lines in the low frequency region. The ohmic resistance (*R*_s_) at the intersection of the curve and the *Z′* axis high frequency region is related to the electrolyte resistance. The semicircle in the intermediate frequency region is related to the charge transfer resistance (*R*_ct_) at the electrode/electrolyte interface, while the straight part represents the Warburg impedance related to the Li^+^ diffusion in the LiFePO_4_ lattice. LFP/C-60 shows the smallest semicircle radius, indicating the smallest charge transfer resistance. The slope of the LFP/C-60 in the straight portion is the largest, indicating improved lithium ion diffusion.

Fig. 5(c) illustrates the galvanostatic charge–discharge curves of LFP/C-0, LFP/C-60 and LFP/C-100 samples at 0.2C. LFP/C-60 has the highest specific capacity of 156.3 mA h g^−1^. This is mainly because the well-coated carbon layer can connect LiFePO_4_ particles and form a good conductive network between the particles, thereby improving the electronic conductivity. The electrode/electrolyte interface area is large, increasing the number of active sites for electrochemical reactions. The discharge capacity of LFP/C-0 sample is below 80 mA h g^−1^. This is because the large particle size results in a long ion transport path. And uneven state of carbon coating is less conducive to electron transport. The potential difference between charging and discharging can reflect the polarization degree of the electrode. The potential difference of LFP/C-0 and LFP/C-100 is 159 mV and 132 mV, respectively. The potential difference of LFP/C-60 is 92 mV, which is the lowest among the three samples. This is due to the uniform carbon coating and twin microspheres structure of LFP/C-60, which ensures the contact and charge transfer between particles.

The cycle stability is an important indicator of the performance of electrode materials. Fig. 5(d) demonstrates the cycle performance of LFP/C-0, LFP/C-60 and LFP/C-100 samples at 1C rate. The discharge capacities of LFP/C-0, LFP/C-60 and LFP/C-100 are 72 mA h g^−1^, 152.2 mA h g^−1^ and 104.9 mA h g^−1^, respectively. The cycle stability of LFP/C-0 and LFP/C-100 deteriorated rapidly, and the capacity loss rate reached 0.07787% and 0.05453% per cycle, accompanied by a decrease in Coulomb efficiency. In contrast, LFP/C-60 has good cycle performance under high coulombic efficiency, and the average capacity loss rate of 1000 cycles is only 0.01737% per cycle.

In order to evaluate the rate performance of the sample, LFP/C-0, LFP/C-60 and LFP/C-100 were tested at different charge and discharge rates. The specific capacity results are shown in Fig. 5(e). LFP/C-60 exhibits the best rate performance. The discharge specific capacities at 0.2C, 0.5C, 1C, 2C, 5C and 10C are 156.3 mA h g^−1^, 151.9 mA h g^−1^, 149.8 mA h g^−1^, 140.2 mA h g^−1^, 129.4 mA h g^−1^ and 118.5 mA h g^−1^, respectively. The discharge capacity is restored to 153.2 mA h g^−1^ when the rate is back to 0.2C. The excellent rate performance of the material is attributed to the following three factors: first, nanoparticles can reduce the diffusion path of lithium ions and improve their rate performance; second, uniform carbon coating can increase the overall electronic conductivity of the material, which is conducive to high rate performance; third, the unique structure and mesoporous voids provide channels for ion transport.

**Fig. 6:**
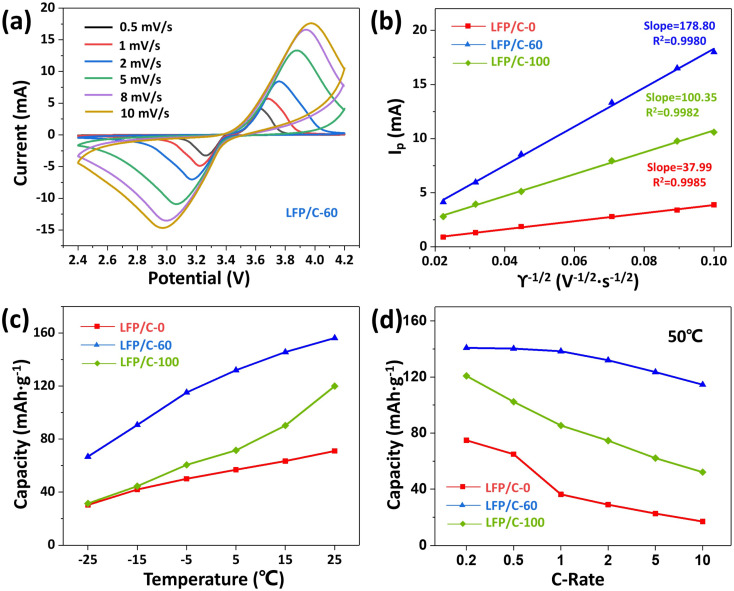
CV curves (a) of LFP/C-60 with different scanning rate from 0.5 mV s^−1^ to 10 mV s^−1^. Linear response of the peak current (*I*_p_) as a function of the square root of scanning rate (*ϒ*) (b), discharge capacity (c) at 0.2C rate under different temperatures, and (d) discharge capacity of LFP/C-0, LFP/C-60, LFP/C-100 at different C-rates under 50 °C.

Fig. 6(a) displays the CV curves of LFP/C-60 at different scan rates (0.5–10 mV s^−1^). It can be seen that the increase of polarization degree of LFP/C-60 with scan rate is the smallest among the three samples, compared with that of LFP/C-0 and LFP/C-100 at different scan rates (0.5–10 mV s^−1^), as shown in Fig. S4(a and b).† In order to further compare the differences in lithium ion diffusion kinetics among the three composites, Fig. 6(b) plots depicted the linear relationship between the peak current and the square root of the scanning speed. The lithium ion diffusion coefficient (*D*_Li^+^_) can be calculated from the Randles–Sevcik equation:^45^

3 *I*_p_ = 269000 × *n*^3/2^·*AC*_0_*D*^1/2^*γ*^1/2^

where *I*_p_ is the peak current (*A*), *n* is the number of electrons transferred by Fe^3+^/Fe^2+^ redox couple, which is determined to be 1, *A* is the electrode surface area (1.33 cm^2^), *C*_0_ is the molar concentration of lithium ion in LiFePO_4_ (7.69 × 10^−3^ mol cm^−3^), *D* is the diffusion coefficient of lithium ion, *γ* is the scanning speed (mV s^−1^).^46^ According to eqn (3), the peak current is linearly related to the square root of the scanning rate. The lithium ion diffusion coefficients of LFP/C-0 and LFP/C-100 are 1.907 × 10^−10^ cm^2^ s^−1^ and 1.330 × 10^−9^ cm^2^ s^−1^, respectively. The lithium ion diffusion coefficient of LFP/C-60 reached 4.228 × 10^−9^ cm^2^ s^−1^, and the ion transport capacity is significantly improved. The results show that the good interface and structure of twin microspheres are beneficial to the diffusion of lithium ions in the electrode, thus enhancing the electrochemical performance of LiFePO_4_/C composites.

Lithium-ion batteries for vehicles require the ability to operate in a wide temperature range. The main challenge of LiFePO_4_ batteries is their poor low-temperature performance. The capacity of LiFePO_4_ will decrease significantly at low temperature, which is related to the decrease of ionic conductivity of lithium salt based electrolyte at low temperature.^47^ On the other hand, the structure of the olivine phosphate cathode material itself determines the slow diffusion of lithium ions in one-dimensional channels, resulting in lower ionic conductivity.^48^ Therefore, shortening the diffusion distance and enhancing the ion transport capacity can significantly improve the performance of LiFePO_4_ at low temperature. As shown in Fig. 6(c), the discharge capacities of LFP/C-0, LFP/C-60 and LFP/C-100 were tested at different temperatures. LFP/C-60 showed higher discharge capacity at low temperatures, even at −15 °C and −25 °C. It can still provide specific capacities of 90.7 mA h g^−1^ and 70.1 mA h g^−1^. This indicates that the fine particles of the twin microspheres shorten the lithium ion transport distance, and the unique structural morphology helps to maintain the rapid transport of ions around the electrode. LFP/C-60 twin microspheres show good discharge ability at room temperature and low temperature, and have the potential to be applied in different climate scenarios.

The discharge capacity at 50 °C under different rate, as depicted in Fig. 6(d), show that the discharge capacity of the three samples is slightly lower than that measured at room temperature. The possible reasons are the inherent properties of LiFePO_4_ and the possible electrolyte decomposition. LFP/C-60 exhibits excellent discharge capacity and retains a discharge capacity of 114.53 mA h g^−1^ even at 10C rate. At 1C rate, the discharge rate of LFP/C-0, LFP/C-60 and LFP/C-100 decreased by 39.7%, 9.07% and 18.6%, respectively compared with the values tested at room temperature. LFP/C-60 exhibits the smallest decline for the high temperature capacity. This means that the existence of the twin microsphere structure enhances the stability of the electrode.

## 4 Conclusion

In summary, twin microspheres LiFePO_4_/C composites were successfully prepared using a mixed solution of certain amount of phosphoric acid and phytic acid as a phosphorus source, which demonstrated the possibility of preparing new complex layered structures without using surfactants. Phytic acid was related to the self-assembly and fusion of particles, while phosphoric acid plays an important bridging role in the assembly process of twin microsphere structures. The configuration of the twin microspheres can be controlled by the relative ratio of phosphoric acid and phytic acid. The introduction of phytic acid can effectively reduce the particle size of primary particles and shorten the distance of lithium ion transport. The uniform carbon coating constructs a conductive network in the twin microspheres structure, which effectively improves the conductivity and interface stability of the electrode material. The retained phytic acid-derived carbon also facilitates to construct a conductive network and reduce electrode polarization. The unique structure and mesoporous channels make the electrolyte easier to infiltrate the electrode material and improving the ionic conductivity of LiFePO_4_/C composites. Therefore, the twin microspheres LiFePO_4_/C composite material has significantly improved the discharge specific capacities and low temperature performance, which can provide higher capacity in a wide temperature range and has potential application value to adapt to various climatic environments.

## Author contributions

The manuscript was written through contributions of all authors. All authors have given approval to the final version of the manuscript.

## Conflicts of interest

The authors declare no competing financial interest.

## Supplementary Material

RA-013-D3RA00183K-s001
