# Supplementary material for: LiFePO4/C twin microspheres as cathode materials with enhanced electrochemical performance
Source: RSC Adv. 2023 Mar 1;13(10):6983–92. doi: 10.1039/d3ra00183k (PMC9977448; doi:10.1039/d3ra00183k)
Supplement: RA-013-D3RA00183K-s001 [file RA-013-D3RA00183K-s001.pdf]

Electronic Supplementary Material (ESI) for RSC Advances.
This journal is © The Royal Society of Chemistry 2023

Supporting Information

# $LiFePO_4$/C Twin Microspheres as Cathode Materials with Enhanced Electrochemical Performance

*Yiqiong Peng, Lingzhi Zeng, Shuai Dai, Feng Liu, Xi Rao*, and Yongping Zhang**

School of Materials and Energy, Southwest University, Chongqing 400715, China

*Corresponding authors, E-mail: zhangyyping6@swu.edu.cn (Y. Zhang), and raoxiemail@swu.edu.cn (X. Rao)

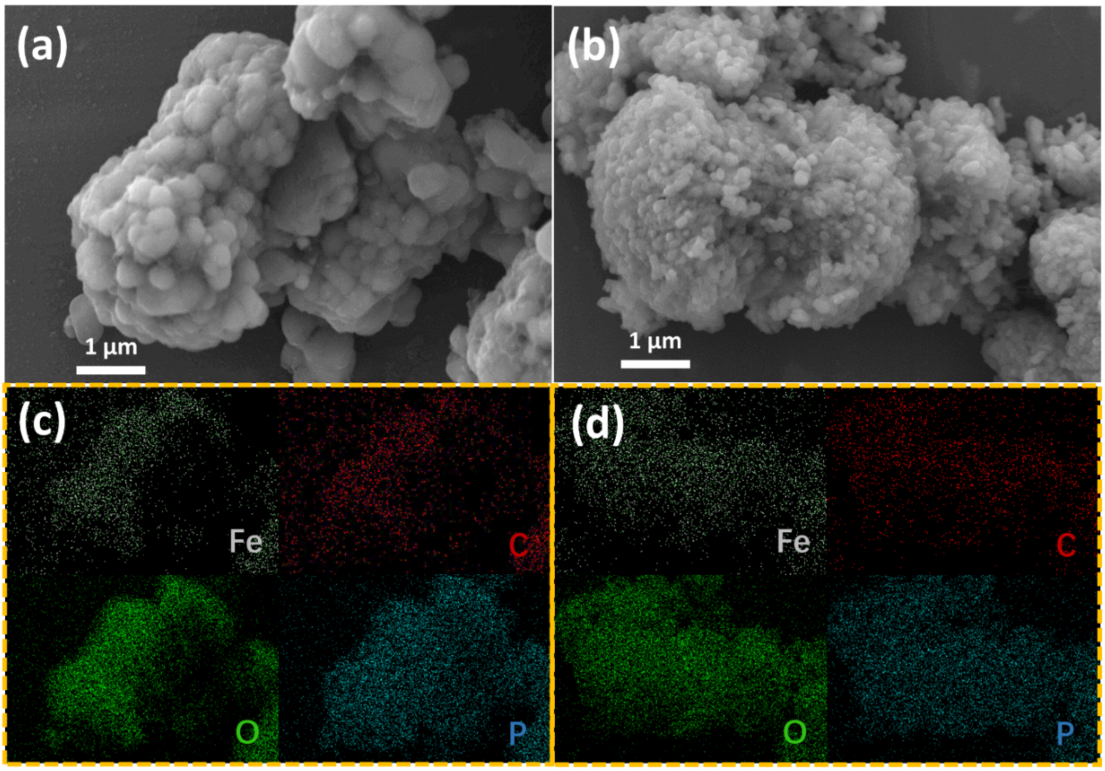

**Fig. S1.** SEM images (a, b) and corresponding EDS mapping (c, d) of LFP/C-40, LFP/C-80;

SEM images in Fig. S2 showed that most of the $LiFePO_4$/C-60 samples appeared as twin microsphere structure with slightly different size. In addition, a small number of twin microspheres were broken during ultrasound process during sample preparation.

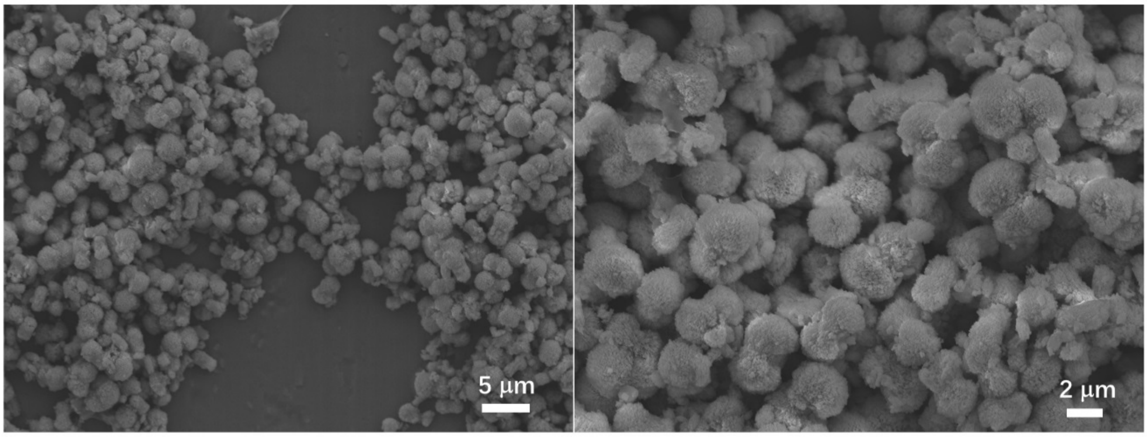


**Fig. S2**. SEM images of $LiFePO_4$/C-60.

The $LiFePO_4$/C-60 twin microspheres present a hierarchical structure assembled by nanoparticles. The hierarchical structure facilitates the rapid and effective transport of matter and charge due to its unique geometric structure[1]. Twin microspheres structures have some advantages. Firstly, the transport of lithium ions in lithium iron phosphate occurs on one-dimensional channels. When there are two or more point defects in the diffusion channel, $Li^+$ cannot enter the material through any side of the tunnel opening, resulting in low utilization of active materials[2]. Nano-sized primary particles promote the transport of $Li^+$ ions by reducing the diffusion distance, improve the utilization of active lithium ions, and smooth the diffusion path to ensure high capacity and rate

capability[2]. Secondly, in practical applications, the low ion diffusion rate is a key factor in the performance of $LiFePO_4$[3]. The porous structure made of uniformly distributed primary particles allows the electrolyte to penetrate finely into the internal active material. The hierarchical structure contributes to the effective contact between $LiFePO_4$ particles and electrolyte, thereby reducing polarization[4]. Thirdly, electrodes made of nanoscale materials are prone to collapse because they cannot be stacked as densely on collectors as micron materials. The micron-sized and robust secondary porous microstructure framework can effectively buffer the aggregation of particles. The micron size and stable secondary porous microstructure can effectively alleviate the aggregation of particles[5]. Fourthly, the large surface area and porous structure of nanomaterials usually result in lower tap density, which leads to lower bulk density of the electrode loaded with nanomaterials [2]. Fourthly, the large surface area and porous structure of nanomaterials usually result in lower tap density, which leads to lower bulk density of the electrode loaded with nanomaterials [6]. In summary, the twin microspheres hierarchical structure integrates the advantages of microsphere particles, porosity, nanocrystallization and carbon coating, ensuring excellent electrochemical performance and good processing performance. Therefore, this twin microsphere structure is a key factor in lithium $LiFePO_4/C$ samples.

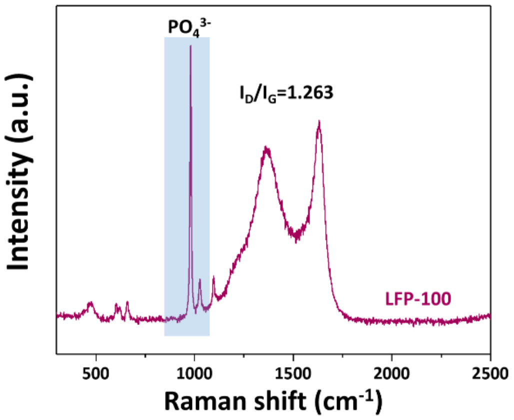


**Fig. S3.** Raman spectrum of LFP-100;

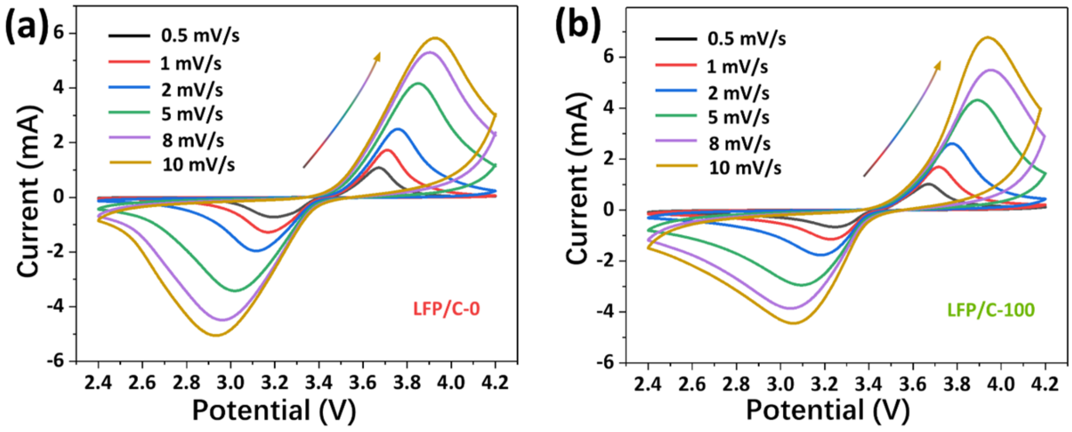


**Fig. S4.** CV curves of LFP/C-0 (a), and LFP/C-100 (b) with different scanning rate between 0.5mV/s and 10mV/s;

High loading increases the ratio of active materials to inactive packaging materials, but it may cause a decrease in discharge capacity[2]. In order to explore the influence of loading amount, the medium (4 mg cm$^{-2}$) and high (8 mg cm$^{-2}$) loading were assembled into button batteries. The discharge capacity was tested at different discharge rates at room temperature, as shown in Fig. S5. The discharge capacity of LFP-60 with medium load and high load reached 146.6 mA·h·g$^{-1}$ and 135.1 mA·h·g$^{-1}$

at 0.2 C discharge rate, while the discharge capacity of LFP-0 is 68.0 mA h $g^{-1}$, 66.3 mA h $g^{-1}$, and the discharge capacity of LFP-100 is 96.9 mA·h·$g^{-1}$, 92.4 mA·h·$g^{-1}$, respectively. The high-loading discharge performance of LFP/C-60 is significantly better than the other two groups of samples. The three-dimensional conductive network constructed in the twin microspheres structure may be the reasons for its excellent performance.

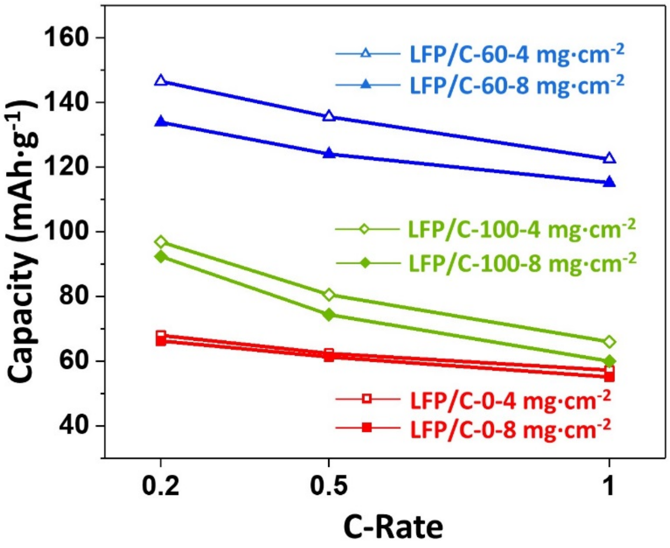


**Fig. S5.** Discharge capacity of LFP/C-0, LFP/C-60, LFP/C-100 at different C-rates with medium and high loading.

References


1. Y. Wu, Z. Wen and J. Li, *Advanced Materials*, 2011, **23**, 1126-1129.
2. Z. Yang, Y. Dai, S. Wang and J. Yu, *Journal of Materials Chemistry A*, 2016, **4**, 18210-18222.
3. J. Hu, W. Huang, L. Yang and F. Pan, *Nanoscale*, 2020, **12**, 15036-15044.
4. Z. Li, J. Yang, T. Guang, B. Fan, K. Zhu and X. Wang, *Small Methods*, 2021, **5**, 2100193.
5. J. Bai, X. Li, G. Liu, Y. Qian and S. Xiong, *Advanced Functional Materials*, 2014, **24**, 3012-3020.
6. H. Wang, L. Liu, R. Wang, X. Yan, Z. Wang, J. Hu, H. Chen, S. Jiang, L. Ni, H. Qiu, H. Tang, Y. Wei, Z. Zhang, S. Qiu and F. Pan, *ChemSusChem*, 2018, **11**,

2255-2261.
